# In-Depth In Silico Search for Cuttlefish (*Sepia officinalis)* Antimicrobial Peptides Following Bacterial Challenge of Haemocytes

**DOI:** 10.3390/md18090439

**Published:** 2020-08-24

**Authors:** Louis Benoist, Baptiste Houyvet, Joël Henry, Erwan Corre, Bruno Zanuttini, Céline Zatylny-Gaudin

**Affiliations:** 1 Normandy University, Unicaen, CNRS, BOREA, 14000 CAEN, France; louis.benoist@unicaen.fr (L.B.); baptiste.satmar@orange.fr (B.H.); joel.henry@unicaen.fr (J.H.); 2 Laboratoire de Biologie des Organismes et Ecosystèmes Aquatiques (BOREA) Université de Caen-Normandie, MNHN, SU, UA, CNRS, IRD, Esplanade de la Paix, CEDEX, 14032 Caen, France; 3 SATMAR, Société ATlantique de MARiculture, Research and Development Department, 50760 Gatteville, France; 4 Plateforme ABiMS, Station Biologique de Roscoff (CNRS-Sorbonne Université), 29688 Roscoff, France; corre@sb-roscoff.fr; 5 Normandy University, Unicaen, Ensicaen, CNRS, GREYC, 14000 Caen, France; bruno.zanuttini@unicaen.fr

**Keywords:** antimicrobial peptide, haemocyte, *Sepia officinalis*, mollusc, challenge, in vitro, *Vibrio splendidus*

## Abstract

Cuttlefish (*Sepia officinalis*) haemocytes are potential sources of antimicrobial peptides (AMPs). To study the immune response to *Vibrio splendidus* and identify new AMPs, an original approach was developed based on a differential transcriptomic study and an in-depth in silico analysis using multiple tools. Two de novo transcriptomes were retrieved from cuttlefish haemocytes following challenge by *V*. *splendidus* or not. A first analysis of the annotated transcripts revealed the presence of Toll/NF-κB pathway members, including newly identified factors such as *So*-TLR-h, *So*-IKK-h and *So*-Rel/NF-κB-h. Out of the eight Toll/NF-κB pathway members, seven were found up-regulated following *V*. *splendidus* challenge. Besides, immune factors involved in the immune response were also identified and up-regulated. However, no AMP was identified based on annotation or conserved pattern searches. We therefore performed an in-depth in silico analysis of unannotated transcripts based on differential expression and sequence characteristics, using several tools available like PepTraq, a homemade software program. Finally, five AMP candidates were synthesized. Among them, NF19, AV19 and GK28 displayed antibacterial activity against Gram-negative bacteria. Each peptide had a different spectrum of activity, notably against *Vibrio* species. GK28—the most active peptide—was not haemolytic, whereas NF19 and AV19 were haemolytic at concentrations between 50 and 100 µM, 5 to 10 times higher than their minimum inhibitory concentration.

## 1. Introduction

Antimicrobial peptides (AMPs) are small peptides commonly ranging from 10 to 50 amino acids in length and displaying activity against bacteria [1,2,3], viruses [4,5], fungi [6,7] or protozoan parasites [8,9]. Most of AMPs are cationic, but some anionic peptides have been identified too [10]. They are secreted by various cell types in a wide diversity of animal species [11], plants [12] and microorganisms [13], and derive from a precursor. Three main types of precursors are described: (1) precursors composed of a signal peptide followed by the mature AMP, like cecropins [14] or penaeidins [15], (2) precursors composed of a propeptide located between the signal peptide and the mature AMP, like cathelicidins [16] or hepcidins [17], or (3) precursors composed of the mature AMP located between the signal peptide and a C-terminal extension, like piscidins [18] or myticalins [19].

Molluscan species only rely on innate immune processes, including secretion of AMPs, to protect themselves against external aggressions [20]. Most of molluscan AMPs have been identified in the haemolymph and/or in the circulating cells—haemocytes (hct)—in bivalve species [1,21,22,23,24,25,26,27,28,29,30]. In many cases, AMPs were identified following bacterial challenge, e.g., mytilin A in the mussel *Mytilus edulis* [30], two isoforms of defensin (*Cg*-defh1 and *Cg*-defh2) [21] and a proline-rich AMP (*Cg*-Prp) [22] in the oyster *Crassostrea gigas*, big defensins *Vp*-BD in the clam *Venerupis philippinarum* [31] and *Sb*-BDefd-1 in the ark clam *Scapharca broughtonii* [28], myticusin-1 in *Mytilus coruscus* [29] and *Pm*AMP-1 in the oyster *Pinctada fucata martensii* [26]. In other cases, differential expression of AMPs was observed following bacterial challenge, like overexpression of defensins in *Mytilus galloprovincialis* [23], big defensins in *C*. *gigas* [24], *Pv*-Def in the mussel *Perna viridis* [25] or downregulation of *Cg*-defh2 in *C*. *gigas* [21] or of penaeidins 3 and 5 in *Fenneropenaeus chinensis* [32]. In Gastropods, a few AMPs have been identified from the haemolymph and/or hct, including a proline-rich peptide from the marine snail *Repana venosa* [33] and defensin from the disk abalone *Haliotis discus discus*, whose expression in hct increased following bacterial challenge [34]. In Cephalopods, no AMP has been identified using classical approaches such as homology search using in silico analysis or immune challenge response. The only two AMPs described were designed in silico using a cuttlefish (*Sepia officinalis*) transcriptomic database including central nervous system, reproductive tissues and posterior salivary glands extracts, but no hct extract [35].

In this context, we studied the immune response of cuttlefish hct. Considering that cuttlefish belongs to animals subjected to directive 2010/63/EU on animal care and protection, an in vitro challenge was developed. The strain used for the challenge was *Vibrio splendidus*, which belongs to the Splendidus clade. Its members are known pathogens of octopus [36] as well as of shellfish hatcheries [37,38] or fish farms [39,40]. In silico analysis of de novo hct transcriptomes did not reveal any AMP based on homology or conserved patterns, so an in-depth in silico study was conducted on the differentially expressed transcripts of control versus challenged hct and AMP precursor characteristics such as the presence of a peptide signal and sequence length.

## 2. Results—Discussion

The two transcriptomes of control hct (c-hct) and *V*. *splendidus*-challenged hct (*Vs*-hct) comprised 96,765 (c-hct) and 107,747 (*Vs*-hct) transcript sequences including isoforms of 88,346 (c-hct) and 98,434 (*Vs*-hct) genes (according to the “gene” definition provided by Trinity assembler), with average lengths of 806.1 (c-hct) and 774.7 (*Vs*-hct) nucleotides, respectively. Based on the presence of a blastx hit against the Uniprot-Swissprot database, 15% and 14% of all transcripts included in the c-hct and *Vs*-hct transcriptomes, respectively, were annotated. These low levels of annotation are striking but commonly observed in such an atypical model, as already reported for the white body (WB) with 20% [41], the central nervous system (CNS) with 28% [42], the accessory nidamental gland (ANG) with 34%, or the posterior salivatory gland (PSG) with 45% [43]. Besides, in another cephalopod species (*Octopus vulgaris*), 18.95% of the hct transcriptome was annotated [44]. These low levels of annotation reflect the lack of available data for cephalopod species and the need for further studies on unannotated transcripts.

Regarding the transcripts per kilobase million (TPM) values obtained during sequencing, the twenty most expressed transcripts (top20) in c-hct corresponded to transferrin, neurofilament, chitin deacetylase, structural polyprotein, polyubiquitin, tropomyosin, filamin-A, dynein light chain, ferritin, matrilin-2 and 3, matrix metalloproteinase, actin, riboflavin kinase, perivitellin, and five unannotated transcripts (Table S1). The top20 in *Vs*-hct corresponded to transferrin, neurofilament, chitin deacetylase, structural polyprotein, filamin-A, polyubiquitin, riboflavin kinase, histone H1, tropomyosin, ferritin, actin, perivitellin, dynein light chain, matrix metalloproteinase, matrilin-3, and five unannotated transcripts (Table S2). Eighteen of the c-hct top20 also belonged to the *Vs*-hct top20. Unknown 5 and matrilin-2 from the c-hct top20 were not part of the *Vs*-hct top20, while histone H1 and unknown 6 from the *Vs*-hct top20 were not in the c-hct top20. Comparing the global fold changes in TPM values between *Vs*-hct and c-hct, 49% of the transcripts seemed up-regulated (fold change > 1.1), 47% seemed down-regulated (fold change < 0.9) and 4% seemed unaffected by the challenge (0.9 ≤ fold change ≤ 1.1). Several transcripts from both top20s were unaffected by the challenge, such as transferrin and chitin deacetylase whose fold changes were 0.98 and 0.93, respectively. Furthermore, four transcripts from both top20s were up-regulated, namely unknowns 1 and 2, riboflavin kinase and perivitelline, with fold changes of 1.66, 1.35, 1.36 and 1.11, respectively. Twelve transcripts from both top20s were down-regulated, namely neurofilament, structural polyprotein, polyubiquitin, filamin-A, ferritin, tropomyosin, unknowns 3 and 4, matrix metalloproteinase, actin, matrilin-3 and dynein light chain, with fold changes of 0.87, 0.72, 0.69, 0.85, 0.60, 0.81, 0.60, 0.84, 0.56, 0.82, 0.56 and 0.78, respectively. The two transcripts only found in the *Vs*-hct top20—histone H1 and unknown 6—were up-regulated by the bacterial challenge, with fold changes of 2.00 and 1.25, respectively. Besides, the two transcripts only found in the c-hct top20—matrilin-2 and -5—were down-regulated, with fold changes of 0.44 and 0.42, respectively (Tables S1 and S2). Interestingly, all unannotated transcripts of the c-hct and *Vs*-hct top20s seemed affected by the challenge with *V*. *splendidus,* highlighting the need for further analysis on unannotated sequences. Among the transcripts from both top20s affected by *V*. *splendidus* exposure, several (polyubiquitin, ferritin, paramyosin and actin) were differentially expressed after bacterial challenge of *Ruditapes decussatus* [45]. Interestingly, actin, which is commonly used as a reference gene, was affected by *V*. *splendidus* exposure, as previously reported for *R*. *decussatus* [45] and *Mya arenaria* [46,47]. The transcript annotated as transferrin coded for a partial sequence sharing sequence identity with salmon (*Oncorhynchus kisutch*) transferrin-like protein (41.16%, P79815.1). Transferrins bind to iron and limit its availability by creating a bacteriostatic environment for iron-dependent bacteria. For example, *Ab*-transferrin was up-regulated upon immune stimulation of *Haliotis discus discus* with *Vibrio parahaemolyticus*, lipopolysaccharides and *Listeria monocytogenes* [48]. Additionally, the transcript coding for a homologue of histone H1 (*So*-H1) was up-regulated after bacterial challenge. Several antibacterial peptides derived from histone H1 have been described in salmon [49] and rainbow trout [50]. *So*-transferrin was highly expressed in both control and challenged conditions, and *So*-H1 expression was two-fold higher after challenge with *V*. *splendidus.* Therefore, it could be involved in the immune response.

Among the transcripts overexpressed in *Vs*-hct, several were involved in the immune response, such as members of the Toll/NF-κB pathway (Table 1).

A Toll-like receptor (TLR) previously identified in ANG and called *So*-TLRγ [51] was also identified in the two hct transcriptomes during this work, together with another sequence corresponding to a partial sequence of a TLR and called *So*-TLR-h. Partial *So*-TLR-h possessed leucine-rich repeat domains, one leucine-rich repeat C-terminal domain, a transmembrane domain and the Toll-interleukin-1 resistance domain, and shared 40.41% sequence identity with TLR-3 from *M*. *corruscus* whose expression was significantly higher in hct, digestive gland and adductor muscle than in foot [52]. Regarding TPM values, *So*-TLRγ and *So*-TLR-h were up-regulated after incubation with *V*. *splendidus,* with fold changes of 1.29 and 15.11, respectively. Several molluscan TLRs were found up-regulated after immune challenge [44,53,54,55,56], confirming their function in the immune response. In addition, one inhibitor of NF-κB kinase (*So*-IKK-h) sharing 27.29% sequence identity with *Cyclina sinensis* IKK (ASE55486.1) and one Rel/NF-κB (*So*-Rel/NF-κB-h) sharing 78.57% sequence identity with *Euprymna scolopes* Rel/NF-κB (AAY27981.1) and possessing the Rel identity domain (RHD) were newly identified in the hct transcriptome. The myeloid differentiation primary response protein MyD88, the interleukin-1 receptor-associated kinase 4 (*So*-IRAK4), the TNF receptor-associated factor 6 (*So*-TRAF6) and the inhibitor of NF-κB (*So*-IκB) previously described in ANG [51] were also identified. All these factors could be involved in signal recognition and transduction leading to activation of immune gene transcription. Except *So*-IκB, all members of the Toll/NF-κB pathway identified in the present study were up-regulated after incubation with *V*. *splendidus*. These results are consistent with those obtained in *M*. *galloprovincialis* where almost all identified members of the Toll/NF-κB pathway were up-regulated after challenge with several bacteria, including *V*. *splendidus* [56].

Immune factors were also identified, such as two peptidoglycan recognition proteins (*So*-PGRP-h1 and *So*-PGRP-h2), one bactericidal permeability-increasing protein/lipopolysaccharide-binding protein (*So*-BPI/LBP), one galectin (*So*-Gal-2) and a partial sequence of inducible nitric oxide synthase (*So*-iNOS-h) (Table 1). NOSs are known to produce nitric oxide (NO), which is involved in various physiological functions but could also kill bacteria when produced at high concentrations. *So*-iNOS-h shared 45.40% sequence identity with *C*. *gigas* NOS (K1QRH7), which was up-regulated in oyster hct after co-stimulation with LPS and TNF-α [57]. Both *So*-PGRP-h1 and *So*-PGRP-h2 identified in the hct transcriptomes possessed the conserved peptidoglycan-binding type-2 amidase domain, characteristic of vertebrate and invertebrate PGRPs. PGRPs are involved in various immune processes such as recognition, signalling and effector function [58]. They were up-regulated in *Argopecten irradians* after exposure to peptidoglycans [59] and in *Chlamys farreri* after exposure to *V*. *anguillarum* and *Micrococcus lysodeikticus* [60]. *So*-BPI/LBP, previously identified in cuttlefish WB [41], shares 52.58% sequence identity with *E*. *scolopes* LBP-3 (AEL03862.1) and 30% sequence identity with *C*. *gigas* LBP/BPI (AAN84552.1), whose recombinant protein possesses antibacterial activity against *Escherichia coli* strains [61]. Finally, partial sequence of *So*-Gal-2 previously identified in cuttlefish WB [41], which shares 46% sequence identity with galectin-2-2 (AJA37869.1) identified in the *Littorina littorea* hct transcriptome [62] was identified in cuttlefish hct. TPM values showed that all these immune-related factors seemed up-regulated with fold change greater than 1.10 (Table 1). Three of them (*So*-Gal-2, *So*-PGRP-h1 and *So*-PGRP-h2) displayed “challenged” TPM values close to or more than 1.5-fold higher than control TPM values. Besides, *So*-iNOS had a TPM value of 0 in the control condition versus 0.52 in the challenged condition, suggesting *V*. *splendidus*-induced expression. All these data tend to confirm that the in vitro challenge performed in this study triggered the immune response of cuttlefish hct.

**Figure 1:**
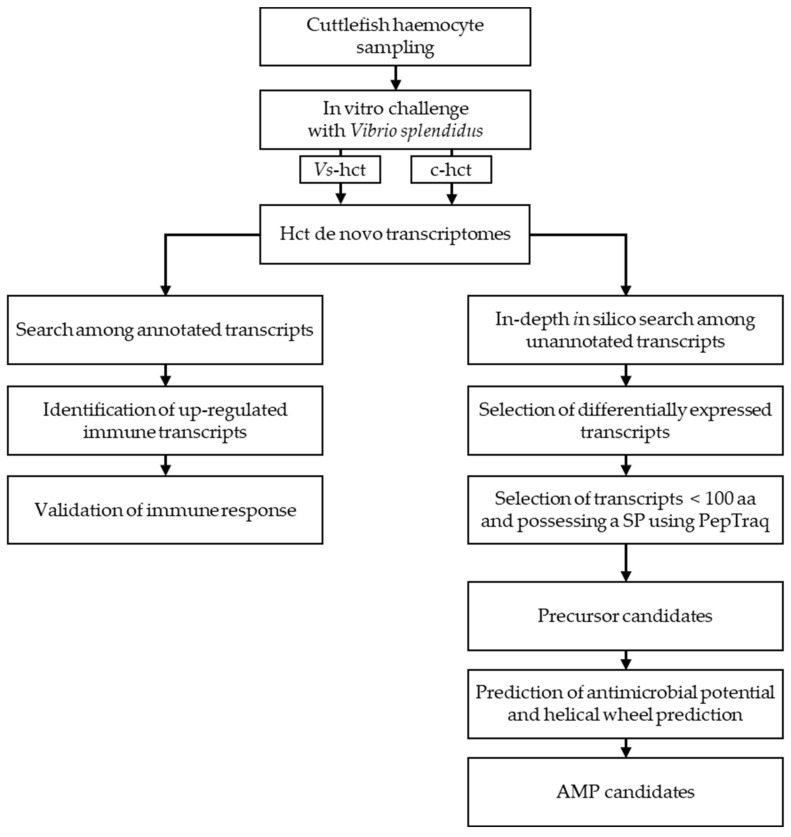
Overview of the methodology used to identify antimicrobial peptides using an in vitro challenge followed by an in-depth in silico search. AMP: antimicrobial peptides.

Our annotation-based search did not identify any AMP, so we performed an in-depth search based on unannotated transcripts, as described in Figure 1.

Among the differentially expressed transcripts between the control and challenged conditions, we selected sequences of less than 100 amino acids presenting a signal peptide, using our homemade PepTraq software program. We applied some of the specific structural AMP criteria previously defined by Houyvet and collaborators [35] and selected five candidates (Table 2).

**Figure 2:**
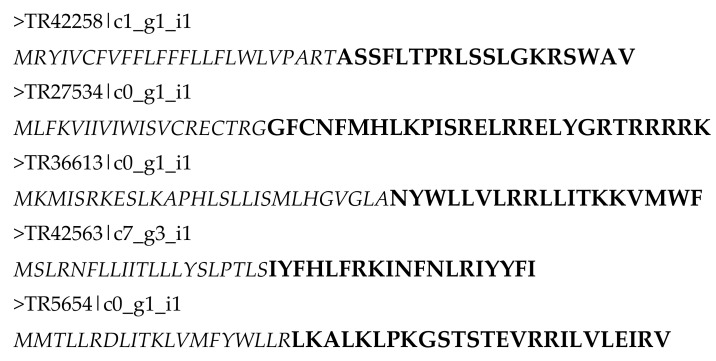
Protein sequences of putative AMP precursors (in italic: predicted signal peptide).

Among them, three were down-regulated after *V*. *splendidus* exposure, with fold changes of 0.39, 0.84 and 0.85. The other two candidates were up-regulated with fold changes of 1.37 and 1.77. Expression values of these transcripts were also obtained for ink sac (male and female), embryo and skin (Table S3). These tissues are known to possess antimicrobial activities, as previously reported in Cephalopods for ink [63] and skin co-products [64,65]. One transcript—TR42563|c7_g3_i1—was almost exclusively expressed in hct. All the other transcripts were expressed in all samples. However, TR27534|c0_g1_i1 was more expressed in hct and skin. The complete sequences of the precursors are presented in Figure 2. The five precursors were between 40 and 54 amino acids in length, and all possessed a signal peptide.

The peptides resulting from the selected precursors were between 19 and 28 amino acids in length, with molecular weights ranging from 2,063.38 to 3,577.25 Da. All peptides were cationic, with net charges ranging between +3 and +8, and hydrophobic ratios ranging between 28% and 63% (Table 3).

**Figure 3:**
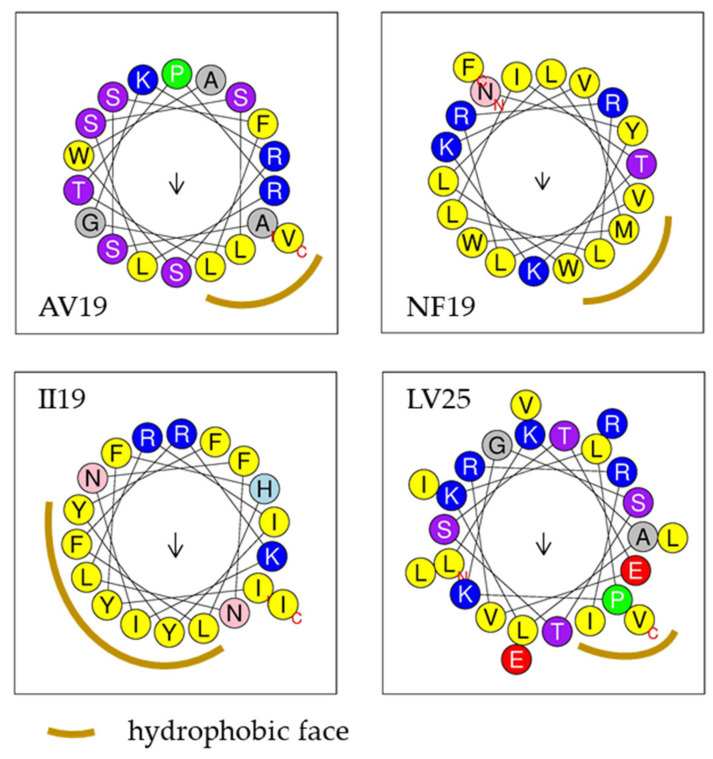
Helical wheel projections of AV19, NF19, II19 and LV25 predicted by the online tool HeliQuest. Yellow: hydrophobic residues; blue: basic residues; green: special residues; red: acidic residues; purple and pink: polar residues; arrow: hydrophobic moment.

Using the Antimicrobial Peptide Database website’s predictor (APD3), four of these five peptides were predicted to form an α-helix possessing 2 to 7 residues in the same hydrophobic surface. Helical wheel projection from HeliQuest demonstrating that these four peptides adopted an α-helix conformation and possessed a hydrophobic face (Figure 3).

The similarity of the five peptides with other known peptides was assayed using APD3 alignment program. AV19 possessed 40% similarity with two frog antibacterial peptides, namely temporin-1Cd from *Rana clamitans* [66] and temporin-1Ola from *R*. *akaloosae* [67]. GK28 possessed 35.48% similarity with marmelittin from the venom of the scorpion *Mesobuthus eupeus* [68] and a C-terminal end similar to the one of GR21 [35], in addition to the lowest hydrophobic ratio and the highest net charge of the selected peptides. Both NF19 and II19 possessed similarity with AMPs from the bovine rumen microbiome—47.36% with P20 and 41.66% with lynronne-2, respectively [69]. Finally, LV25 possessed 37.93% similarity with human peptide 6 [70].

The antimicrobial potential of the five peptides was assayed on ten bacteria—8 Gram-negative ones and 2 Gram-positive ones—i.e., *V*. *splendidus*, a cuttlefish pathogen *V*. *alginolyticus* [71], other aquatic pathogens *V*. *anguillarum* [72], *Aeromonas salmonicida* [73], *Lactococcus garvieae* [74] and *V*. *parahaemolyticus* causing human seafood-related illness [75]. Among the five peptides tested, three—AV19, GK28 and NF19—were active against at least one bacterial species (Table 4), whereas II19 and LV25 were not active against any bacterial species. None of the five peptides was active against Gram-positive bacteria. The three active peptides were all active against *V*. *splendidus*. NF19 inhibited *V*. *splendidus* growth between 5 and 10 µM and was bactericidal between 10 and 20 µM. While its closest homologue P20 from the rumen microbiome was only semi-active against epidemic meticillin-resistant *Staphylococcus aureus* (EMRSA-15) [69], NF19 was strongly active against *V*. *splendidus* but did not show any activity against Gram-positive bacteria. AV19 inhibited both *V*. *splendidus* and *V*. *parahaemolyticus* between 10 and 20 µM and was bactericidal between 10 and 20 µM and 20 and 50 µM, respectively. Both frog temporins 1-Cd and 1Ola—the closest homologues of AV19—were active against *S*. *aureus*, and also against *Bacillus subtilis* for temporin-1Ola, but not against *E*. *coli* [66,76], showing possible Gram-positive selectivity. As for AV19, it seemed active only against some *Vibrio* species.

Only one peptide—GK28—was active against the five *Vibrio* species. GK28 inhibited the growth of *V*. *alginolyticus*, *V*. *splendidus* and *V*. *parahaemolyticus* equally as the positive control oxytetracycline did at concentrations ≤5 µM and was also equally bactericidal against *V*. *splendidus*. In contrast to marmelittin—its closest homologue active against a broad range of Gram-negative and Gram-positive bacteria [68]—GK28 seemed only active against Gram-negative bacteria. GK28 was the only peptide active against a non-*Vibrio* species, i.e., *E*. *coli*. None of the five peptides was active against the other two Gram-negative bacteria *Halomonas aquamarina* and *A*. *salmonicida* (Table 4). The potential synergistic antibacterial activity of the active peptides and of the two up-regulated peptides—GK28 and LV25—was assayed. No synergistic activity was observed between the two most expressed peptides. Neither NF19 nor AV19 improved the antimicrobial activity of GK28. However, while AV19 and NF19 only delayed *V*. *alginolyticus* growth when tested alone (data not shown), a synergistic activity was observed when they were tested together (Table 4). No synergistic activity of NF19 and AV19 was observed against *V*. *splendidus*.

**Figure 4:**
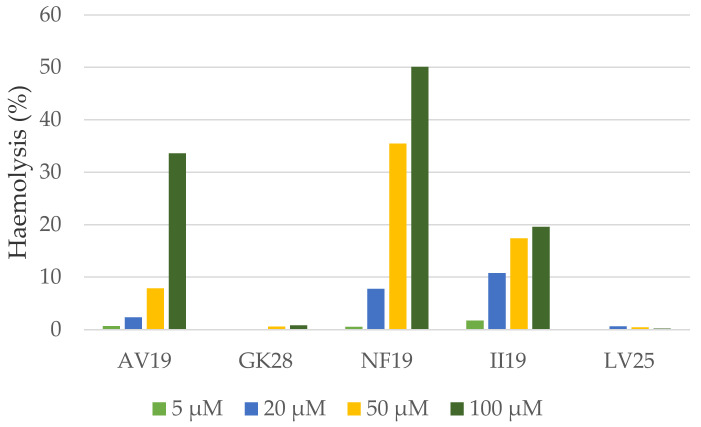
Haemolytic activity of the selected peptides on human red blood cells.

To test the toxicity of the newly identified peptides on eukaryotic cells, their haemolytic activity was assayed on human red blood cells (Figure 4).

At the lowest concentration tested—5 µM—, no haemolytic activity was observed for any of the five peptides. GK28 and LV25 were not haemolytic at any of the concentrations tested. AV19 was slightly haemolytic at 5, 20 and 50 µM, and induced 33% haemolysis at 100 µM. II19 induced 11%, 17% and 19% haemolysis at 20, 50 and 100 µM, respectively. Finally, NF19 was the most haemolytic peptide: it induced 35 and 50% haemolysis at 50 and 100 µM, respectively. The most active peptide—GK28—was not haemolytic at any concentration whereas its closest homologue marmelittin is highly haemolytic on mouse, bird and lizard erythrocytes [68].

Three peptides possessing antibacterial activity were identified. NF19, AV19 and GK28 seemed to possess a complementary antibacterial activity, as observed with mussel myticalins [19]. Such complementarity may reflect the biological role of these AMPs in the cuttlefish immune response and need to be confirmed by in situ analysis. Out of the three active peptides, two were down-regulated by bacterial challenge and one was up-regulated. AMP down-regulation was previously observed in *C*. *gigas* hct: *Cg*-defh2 was down-regulated 24 h and 48 h post challenge with a heat-killed mix of *Micrococcus luteus*, *V*. *splendidus* and *V*. *anguillarum,* but up-regulated in the mantle and gills [21]. In *M*. *galloprovincialis*, myticin and mytilin were found down-regulated in hct after injection of heat-killed *V*. *splendidus,* while defensin was up-regulated. Moreover, after injection of heat-killed *V*. *anguillarum*, only mytilin was up-regulated while defensin and myticin seemed unaffected [77]. Similar results were obtained in shrimp in which Pen3 and Pen5 were down-regulated 24 h after injection of heat-killed *V*. *anguillarum,* while crustin and antilipopolysaccharide factor (ALF) were up-regulated [32]. Again, this may reflect the complementary activity of AMPs in the immune response. Finally, it is also interesting to note that the time lapse between bacterial exposure and the regulation of expression in hct varies greatly according to the AMP. For example, some AMPs are regulated rapidly: *M*. *galloprovincialis* defensin was up-regulated from 1 h after injection of heat-killed *V*. *splendidus* [77], *Pinctada martensii* AMP-1 was up-regulated 2 h after injection of a heat-killed mix of *E*. *coli* and *M*. *luteus* [26], or *Scapharca broughtonii Sb*-BDef1 was up-regulated 8 h and 16 h after injection of *V*. *anguillarum* [28]. Other AMPs are up-regulated late: *M*. *coruscus* myticusin-1 expression peaked 36 h after injection of heat-killed *E*. *coli* [29], or *C*. *gigas Cg*-BigDef1 was up-regulated 24 h, 48h and 72 h after injection of *V*. *splendidus* and *Vibrio tasmaniensis* [24]. It would be interesting to study the time-course of the relative expression of the three AMPs NF19, AV19 and GK28 during bacterial challenge with different bacteria.

## 3. Materials and Methods

### 3.1. Animals

Healthy cuttlefish (*Sepia officinalis*) were caught in the Bay of the Seine between January and June 2015. They were maintained in 1000-L outflow tanks with a permanent water supply at the Centre de Recherches en Environnement Côtier (CREC, marine station of the University of Caen, Luc-sur-Mer, France). Water temperature was maintained at 16 °C.

### 3.2. Ethical Statement

This research followed the guidance given by Directive 2010/63/EU and French regulations regarding the use of animals for experimental procedures. It was approved by the Regional Ethical Committee Cenomexa (Committee agreement number 54; project agreement number 03145.03). The experiment was designed to decrease animal distress by minimising the number of animals. Moreover, the tanks were modified so as to provide hiding places for cuttlefish.

### 3.3. Haemolymph Collection and Challenge with Heat-Killed Bacteria

Two female cuttlefish were anesthetised with ethanol 3% before tissue collection. Haemolymph was withdrawn from the vena cava and branchial hearts using a 10 mL syringe. *Vibrio splendidus* strain CIP 107,715 was grown under agitation at 20 °C in Marine Broth (Conda, 40.20 g/L) medium for 24 h. The culture was centrifuged (4,000× *g*, 5 min, room temperature), washed twice, and resuspended in sterile sea water to an optical density at 600 nm (OD_600_) of 1 (1 × 10^9^ CUF/mL).

Haemolymph was diluted in sterile sea water (*v*/*v*) and divided into two batches: 10 mL remained untreated, and 10 mL were challenged with 200µl of boiled bacteria (boiled 15 min at 100 °C). Haemolymph with and without heat-killed bacteria was then incubated at 16 °C at 100 rpm in a MaxQ6000 incubator (ThermoFisher) equipped with an orbital shaker. After 24 h, hct from the two conditions were harvested by centrifugation at 12,000 rpm and resuspended in TriReagent (Sigma-Aldrich, Saint Louis, MO, USA) to extract total RNA.

### 3.4. Illumina Sequencing

RNA extractions were performed as described by Cornet and collaborators [43]. For sequencing, other samples than hct were used, namely ink sac, skin, and embryo. Total RNA concentrations were quantified using a NanoDrop spectrophotometer (ThermoFisher), and RNA quality was checked using a Bioanalyzer (Agilent Technologies). Library preparation and sequencing was conducted by the McGill University and Génome Québec Innovation Centre (Montréal, Québec, Canada) following the manufacturer’s instructions (Illumina, San Diego, CA). The detailed protocol is available in the supporting experimental section.

Raw data are accessible under ENA project PRJEB39162.

### 3.5. Bioinformatic Analysis

#### 3.5.1. Transcriptome Assembly

Quality checks of raw data from the six libraries were performed with FastQC v. 0.11.5 (https://www.bioinformatics.babraham.ac.uk/projects/fastqc/). Low quality trimming was performed with Trimmomatic v. 0.36 [78] using the following parameters: leading 3, trailing 3, sliding window 4:15, minlen 30. Reads from all tissues were assembled de novo all together with Trinity v 2.2.0 [79] including the read normalisation step corresponding to the Trinity implementation of the diginorm method. The assembler was run with default parameters. The sequences from each library were aligned with RSEM [80] against de novo assembly.

#### 3.5.2. Transcriptome Annotation

Peptide prediction was performed using Transdecoder 5.2.0 [81]. Similarity search (diamond v0.7.9) [82] of the Transdecoder predicted peptides was performed against the Uniprot-Swissprot databases and UniRef90 (release 2018). Peptide signal prediction was performed using SignalP v4.1 [83]. Transmembrane peptide detection was performed using TMHMM v2.0c [84]. Protein domain search was performed using hmmscan from the hmmer v.3.1b1 suite against the Pfam-A database (release 31.0) [85]. Finally, transcriptome functional annotation was performed using the Trinotate pipeline (http://trinotate.github.io described) [81]. An additional annotation-based search was conducted on the hct transcriptomes (c-hct and *Vs*-hct) searching for relevant immune-related transcripts and AMPs. Specific domains were predicted using the online tool SMART (http://smart.embl-heidelberg.de/) [86].

#### 3.5.3. PepTraq

PepTraq is a homemade software program developed to perform in silico analyses from “-omic” data (https://peptraq.greyc.fr). The search for precursors or peptides through PepTraq can be achieved using several structural criteria, as described by Zatylny-Gaudin and collaborators [42] for neuropeptides or by Houyvet and collaborators [35] for antibacterial peptides. The PepTraq version used for this work included new features with respect to previous ones, in particular (1) the prediction of Sec-dependent signal peptides, using package JSPP [87] and (2) the selection of sequences containing a certain number of occurrences of a given pattern (possibly including a number of unspecified amino acids).

#### 3.5.4. In-Depth In Silico Search

In addition to an annotation-based search, we performed an in-depth in silico search using our homemade software PepTraq to look for AMPs. PepTraq is a useful tool developed to conduct in silico approaches on transcriptomic or proteomic datasets based on specific criteria (e.g., the presence of a signal peptide, sequence length, the occurrence and number of certain amino acids or specific patterns, the occurrence of consensus subsequences, the hydrophobicity degree, the electrical charge of protein sequences). The pipeline of the methodology used during this work is illustrated in Figure 1. We targeted unannotated transcripts differentially expressed between control and challenged conditions as well as the length of the resulting precursors and the presence of signal peptides. Among the selected precursors, we targeted some AMP characteristics, as previously described by Houyvet and collaborators [35]. The antimicrobial potential of the selected peptides was evaluated using the CAMP database website’s prediction tool [88] (http://www.camp.bicnirrh.res.in/index.php). We selected the following four algorithms: Support Vector Machine (SVM), Random Forests (RF), Artificial Neural Network (ANN) and Discriminant Analysis (DA). The molecular weight (MW), the net charge, the hydrophobic ratio (HR), α-helix prediction and the number of residues of the same hydrophobic surface of the selected peptides were calculated with the Antimicrobial Peptide Database (APD) website’s calculator and predictor (APD3) [89] (http://aps.unmc.edu/AP/prediction/prediction_main.php). The helical wheel projection diagrams were predicted using Heliquest website’s sequence analysis module [90] (https://heliquest.ipmc.cnrs.fr/cgi-bin/ComputParams.py).

### 3.6. Peptide Synthesis

The five selected peptides were synthesized by GENECUST (Dudelange, Luxembourg) with >95% overall purity.

### 3.7. Antimicrobial Assay

The minimal growth inhibitory concentrations (MICs) of synthetic peptides (Genecust) were evaluated on *Vibrio alginolyticus* (CIP 109819), *V*. *splendidus* (CIP 107715), *V*. *aestuerianus* (CIP 102971), *V*. *anguillarum* (CIP 64.14), *V*. *parahaemolyticus* (CIP 73.30), *Halomonas aquamarina* (CIP 105454T), *Escherichia coli* (CIP 54.8T), *Aeromonas salmonicida* (CIP 103209T), *Enteroccocus faecalis* (CIP 76.117) and *Lactococcus garvieae* (CIP 104369) obtained from the “Collection de l’Institut Pasteur”. Bacteria were cultured in adapted media: Homemade marine broth (HMB, sea salts 20 g/L, peptone 5 g/L, yeast extract 3 g/L) for *Vibrio* species and *H*. *aquamarina*; Luria-Bertani (peptone 10 g/L, yeast extract 5 g/L, NaCl 10 g/L) for *E*. *faecalis* and *E*. *coli*; Trypticasein Soy Broth (TSB, Conda, 40 g/L) for *A*. *salmonicida,* and Brain Heart Infusion (BHI) (Conda, 37 g/L) for *L*. *garvieae*. MICs were determined in triplicate by liquid growth inhibition assays adapted from Hetru and Bulet [91]. Briefly, 10 µL of peptide solution were incubated in Bioscreen honeycomb-well plates with 90 µl of bacterial suspension at a starting OD_600_ of 0.001 in BHI for *L*. *garvieae*, poor broth (peptone 10 g/L, NaCl 5 g/L) for *E*. *faecalis* and E. *coli*, TSB for *A*. *salmonicida*, HMB for *H*. *aquamarina* and saline poor broth (peptone 10 g/L, NaCl 15 g/L) for *Vibrio* species. Bacterial growth was monitored for 16 h by measuring OD_600_ values every 5 min (Bioscreen C, Labsystem, Finland). The peptides were tested alone at concentrations of 5 µM, 10 µM, 20 µM and 50 µM. For synergy, peptides pairs were tested at concentrations of 5 µM, 10 µM and 20 µM. Oxytetracycline at 5 µM and 10 µM was used as a positive control, and water was used as a blank. MICs were expressed in µM as an [a]–[b] concentration interval where [a] was the last concentration with bacterial growth and [b] was the first concentration with 100% bacterial growth inhibition. MBCs (minimal bactericidal concentrations) were expressed in µM as an [a]–[b] concentration interval where [a] was the last concentration with bacterial growth and [b] was the first concentration that killed 100% of the bacteria [91].

### 3.8. Haemolytic Assay

The haemolytic activity of the synthetic peptides was determined in triplicate on human red blood cells (RBCs) as described by Duval and collaborators [92]. Briefly, RBCs were pelleted and washed three times in phosphate-buffered saline (PBS). Then, they were resuspended in PBS to obtain a 1% solution of RBC. Ten microliters of peptide solution were incubated with 90 µL of the RBC 1% solution for 1 h at 37 °C. The peptides were diluted in PBS and tested at different concentrations: 5 µM, 20 µM, 50 µM and 100 µM. Haemolytic activity was determined by measuring the OD at 415 nm. Zero haemolysis and total (100%) haemolysis were determined with PBS and 1% Triton X-100 solutions, respectively.

## 4. Conclusions

In vitro challenge with *Vibrio splendidus* was sufficient to trigger the immune response and the overexpression of members of the Toll/NF-κB pathway and other immune-related factors in cuttlefish haemocytes. As no annotated AMP was identified, we carried out an in-depth in silico search with PepTraq, based on specific characteristics and expression values between control and challenge conditions. This stringent methodology allowed us to select a small list of candidates, 60% of which displayed antibacterial activity. Three peptides displayed targeted activity against *Vibrio* species, and one of them—GK28—was active at low concentrations and did not induce haemolysis of human red blood cells even at high concentrations. Considering that these three peptides were identified from a precursor with a signal peptide, it would be interesting to validate their biological function in cuttlefish. The methodology used in this study, based on Houyvet and collaborators [35] as an alternative to classical methods requiring a lot of biological material, was focused on the reduction of animal pain and use, consistent with directive 2010/63/EU on animal care and protection.

## Figures and Tables

**Table 1:** Immune-related transcripts identified in the hct transcriptomes.

Transcript	Name	Transcript Length (nt)	Protein Length (aa)	Expression (TPM)		Fold Change
				c-hct	*Vs*-hct	
TR5906|c0_g1_i2	TLRγ	2861	833	3.55	**4.58**	1.29
TR31922|c0_g2_i3	TLR-h (partial)	1741	536	0.31	**4.71**	15.11
TR20767|c0_g1_i1	MyD88	1938	338	12.13	**14.50**	1.20
TR42010|c1_g1_i1	IRAK4	1939	319	8.56	**10.63**	1.24
TR37884|c0_g2_i2	TRAF6	2364	547	2.53	**3.05**	1.21
TR17535|c0_g1_i1	IκB	2609	337	233.96	230.08	0.98
TR34670|c0_g1_i1	IKK-h	2724	476	10.64	**12.29**	1.16
TR41212|c6_g1_i2	Rel/NF-κB-h	2614	491	1.85	**2.56**	1.39
TR24628|c0_g1_i1	iNOS-h (partial)	4137	1106	0.00	**0.52**	-
TR41722|c24_g56_i1	PGRP-h1	873	213	254.12	**466.71**	1.84
TR14900|c0_g1_i1	PGRP-h2	956	204	87.28	**154.73**	1.77
TR40338|c3_g2_i1	BPI/LPB	2342	537	134.38	**149.31**	1.11
TR32087|c0_g2_i1	Gal-2 (partial)	817	236	86.15	**120.87**	1.40

aa: amino acid, nt: nucleotide, TPM: transcripts per kilobase million.

**Table 2:** Putative AMP precursors identified in the hct transcriptomes.

Transcript Name	Length (aa)	Expression (TPM)		Fold Change	SP	AMP Name
		c-hct	*Vs*-hct			
TR42258|c1_g1_i1	54	**8.92**	7.57	0.85	Yes	AV19
TR27534|c0_g1_i1	48	17.39	**23.79**	1.37	Yes	GK28
TR36613|c0_g1_i1	47	**20.28**	17.01	0.84	Yes	NF19
TR42563|c7_g3_i1	40	**1.99**	0.78	0.39	Yes	II19
TR5654|c0_g1_i1	45	9.77	**17.33**	1.77	Yes	LV25

aa: amino acid, TPM: transcripts per kilobase million, SP: signal peptide.

**Table 3:** Peptide characteristics of the selected candidates.

Name	Sequence	Length (aa)	MW	C	HR	α-Helix Prediction and NRH	CAMP Algorithms			
							SVM	RFC	ANN	DAC
AV19	ASSFLTPRLSSLGKRSWAV	19	2063.38	+3	42%	Yes-2	0.75	0.8	AMP	0.91
GK28	GFCNFMHLKPISRELRRELYGRTRRRRK	28	3577.25	+8	28%	No	0.69	0.31	AMP	0.8
NF19	NYWLLVLRRLLITKKVMWF	19	2493.14	+4	63%	Yes-7	0.43	0.71	AMP	0.65
II19	IYFHLFRKINFNLRIYYFI	19	2581.1	+3	52%	Yes-6	0.13	0.65	AMP	0.87
LV25	LKALKLPKGSTSTEVRRILVLEIRV	25	2820.45	+4	44%	Yes-4	0.68	0.88	NAMP	0.84

aa: amino acid, MW: molecular weight (Dalton), C: charge, HR: hydrophobic ratio, NRH: number of residues in the same hydrophobic surface, SVM: Support vector machine classifier, RFC: random forest classifier, ANN: artificial neural network, DAC: discriminant analysis classifier.

**Table 4:** Antimicrobial activity of the synthetic peptides.

Name	Gram Negative																Gram Positive			
	*Vibrio alginolyticus*		*Vibrio splendidus*		*Vibrio aestueranius*		*Vibrio anguillarum*		*Vibrio parahaemo-lyticus*		*Escherichia coli*		*Halomonas aquamarina*		*Aeromonas salmonicida*		*Enterococcus faecalis*		*Lactococcus garvieae*	
	MIC	MBC	MIC	MBC	MIC	MBC	MIC	MBC	MIC	MBC	MIC	MBC	MIC	MBC	MIC	MBC	MIC	MBC	MIC	MBC
AV19	-	-	10–20	10–20	-	-	-	-	10–20	20–50	-	-	-	-	-	-	-	-	-	-
GK28	≤5	20–50	≤5	≤5	10–20	>50	10–20	20–50	≤5	10–20	≤5	20–50	-	-	-	-	-	-	-	-
NF19	-	-	5–10	10–20	-	-	-	-	-	-	-	-	-	-	-	-	-	-	-	-
II19	-	-	-	-	-	-	-	-	-	-	-	-	-	-	-	-	-	-	-	-
LV25	-	-	-	-	-	-	-	-	-	-	-	-	-	-	-	-	-	-	-	-
SYN	10–20	>20	5–10	10–20	-	-	-	-	-	-	-	-	-	-	-	-	-	-	-	-
Ox	≤5	≤5	≤5	≤5	≤5	≤5	≤5	≤5	≤5	≤5	≤5	≤5	>10	-	≤5	≤5	≤5	5–10	≤5	> 10

## Supplementary Materials

The following are available online at https://www.mdpi.com/1660-3397/18/9/439/s1, Supporting experimental section. Table S1: Twenty most expressed transcripts in c-hct, Table S2: Twenty most expressed transcripts in Vs-hct. Table S3: Expression patterns of selected transcripts.
