# Supplementary material for: In-Depth In Silico Search for Cuttlefish (Sepia officinalis) Antimicrobial Peptides Following Bacterial Challenge of Haemocytes
Source: Mar Drugs. 2020 Aug 24;18(9):439. doi: 10.3390/md18090439 (PMC7551771; doi:10.3390/md18090439)
Supplement: Supplementary file 1 [file marinedrugs-18-00439-s001.pdf]

Supporting information

In-depth in silico search for cuttlefish (Sepia officinalis) antimicrobial peptides following bacterial challenge of haemocytes.

Louis Benoist[1,2], Baptiste Houyvet[1,2,5], Joël Henry[1,2], Erwan Corre[4], Bruno Zanuttini[3] and Céline Zatylny-Gaudin[1,2,*]

[1] NORMANDIE UNIV, UNICAEN, CNRS, BOREA, 14000 CAEN, France
[2] Laboratoire de Biologie des Organismes et Ecosystèmes Aquatiques (BOREA) Université de Caen-Normandie, MNHN, SU, UA, CNRS, IRD, Esplanade de la Paix, 14032 Caen Cedex, France
[3] Normandie Univ., UNICAEN, ENSICAEN, CNRS; GREYC, 14 000 Caen, France
[4] Plateforme ABiMS, Station Biologique de Roscoff (CNRS-Sorbonne Université), 29688 Roscoff, France
[5] SATMAR, Société ATlantique de MARiculture, Research and Development Department, Gatteville, France

*Corresponding author: celine.gaudin@unicaen.fr

Supporting experimental section **2**

Supplemental Tables:

Table S1. Twenty most expressed transcripts in c-hct 3

Table S2. Twenty most expressed transcripts in *Vs*-hct 4

Table S3. Expression pattern of selected transcripts 5

Supporting experimental section

Detailed quality control and cDNA library preparation protocols

Total RNA was quantified using a NanoDrop Spectrophotometer ND-1000 (NanoDrop Technologies, Inc.), and its integrity was assessed on a 2100 Bioanalyzer (Agilent Technologies). Libraries were generated from 250 ng of total RNA as follows: mRNA enrichment was performed using the NEBNext Poly(A) Magnetic Isolation Module (New England BioLabs). cDNA synthesis was achieved using NEBNext RNA First Strand Synthesis and NEBNext Ultra Directional RNA Second Strand Synthesis Modules (New England BioLabs). The remaining steps of library preparation were performed using NEBNext Ultra II DNA Library Prep Kit for Illumina (New England BioLabs). Adapters and PCR primers were purchased from New England BioLabs. Libraries were quantified using the Quant-iT™ PicoGreen® dsDNA Assay Kit (Life Technologies) and the Kapa Illumina GA with Revised Primers-SYBR Fast Universal kit (Kapa Biosystems). Average size fragment was determined using a LabChip GX (PerkinElmer) instrument.

Supplementary Tables:

Table S1. Twenty most expressed transcripts in c-hct (°: c-hct-specific transcripts)

	Name	Transcript	Expression (TPM)		Fold change
			c-hct	*Vs*-hct	
1	Transferrin	TR90372|c1_g1_i1	28022.57	27562.43	0.98
2	Neurofilament	TR19485|c1_g1_i1	**26823.13**	23315.72	0.87
3	Chitin deacetylase	TR33375|c0_g1_i1	16019.63	14894.69	0.93
4	Structural polyprotein	TR42824|c0_g1_i1	**14678.55**	10635.02	0.72
5	Polyubiquitin	TR38732|c0_g23_i1	**7339.71**	5075.77	0.69
6	Filamin-A	TR33303|c3_g1_i1	**6632.45**	5638.52	0.85
7	Unknown 1	TR41826|c5_g19_i2	6351.18	**10524.84**	1.66
8	Ferritin	TR34156|c1_g1_i1	**5373.48**	3223.95	0.60
9	Unknown 2	TR39529|c1_g1_i1	4465.00	**6042.63**	1.35
10	Tropomyosin	TR38640|c3_g1_i7	**4452.91**	3596.50	0.81
11	Unknown 3	TR42689|c4_g10_i1	**4086.45**	2463.57	0.60
12	Matrix metalloproteinase	TR35643|c1_g1_i1	**3933.39**	2220.67	0.56
13	Matrilin-2°	TR5676|c0_g1_i1	**3770.58**	1647.49	0.44
14	Actin	TR37733|c5_g10_i2	**3672.30**	2996.36	0.82
15	Matrilin-3	TR42669|c4_g1_i1	**3612.10**	2030.77	0.56
16	Riboflavin kinase	TR58606|c1_g1_i1	3441.99	**4689.02**	1.36
17	Dynein light chain	TR42526|c19_g12_i4	**3329.41**	2603.44	0.78
18	Unknown 4	TR36711|c0_g3_i2	**2752.47**	2320.78	0.84
19	Unknown 5°	TR74858|c0_g1_i1	**2630.99**	1100.13	0.42
20	Perivitellin	TR38132|c1_g1_i1	2420.55	**2693.39**	1.11

Table S2. Twenty most expressed transcripts in Vs-hct (*: Vs-hct-specific transcripts)

	Name	Transcript	Expression (TPM)		
			c-hct	*Vs*-hct	Fold change
1	Transferrin	TR90372|c1_g1_i1	28022.57	27562.43	0.98
2	Neurofilament	TR19485|c1_g1_i1	**26823.13**	23315.72	0.87
3	Chitin deacetylase	TR33375|c0_g1_i1	16019.63	14894.69	0.93
4	Structural polyprotein	TR42824|c0_g1_i1	**14678.55**	10635.02	0.72
5	Unknown 1	TR41826|c5_g19_i2	6351.18	**10524.84**	1.66
6	Unknown 2	TR39529|c1_g1_i1	4465.00	**6042.63**	1.35
7	Filamin-A	TR33303|c3_g1_i1	**6632.45**	5638.52	0.85
8	Polyubiquitin	TR38732|c0_g23_i1	**7339.71**	5075.77	0.69
9	Riboflavin kinase	TR58606|c1_g1_i1	3441.99	**4689.02**	1.36
10	Histone H1*	TR41094|c2_g12_i1	2280.01	**4563.54**	2.00
11	Tropomyosin	TR38640|c3_g1_i7	**4452.91**	3596.50	0.81
12	Ferritin	TR34156|c1_g1_i1	**5373.48**	3223.95	0.60
13	Actin	TR37733|c5_g10_i2	**3672.30**	2996.36	0.82
14	Perivitellin	TR38132|c1_g1_i1	2420.55	**2693.39**	1.11
15	Unknown 6*	TR38730|c4_g1_i1	2123.73	**2653.98**	1.25
16	Dynein light chain	TR42526|c19_g12_i4	**3329.41**	2603.44	0.78
17	Unknown 3	TR42689|c4_g10_i1	**4086.45**	2463.57	0.60
18	Unknown 4	TR36711|c0_g3_i2	**2752.47**	2320.78	0.84
19	Matrix metalloproteinase	TR35643|c1_g1_i1	**3933.39**	2220.67	0.56
20	Matrilin-3	TR42669|c4_g1_i1	**3612.10**	2030.77	0.56

Table S3. Expression patterns of selected transcripts. (ED: embryo, ISF: ink sac female, ISM: ink sac male, S: skin).

**Transcript ID**	**Expression (TPM)**					
	***Vs*-hct**	**c-hct**	**EB**	**ISF**	**ISM**	**S**
TR42258|c1_g1_i1	7.566	8.928	36.498	4.556	3.755	23.107
TR27534|c0_g1_i1	23.786	17.388	3.984	1.758	1.58	10.381
TR36613|c0_g1_i1	17.011	20.28	19.871	5.199	6.307	20.806
TR42563|c7_g3_i1	0.775	1.992	0	0	0	0.319
TR5654|c0_g1_i1	17.331	9.773	59.912	42.483	30.16	61.859
